# A therapeutic vaccine prototype induces protective immunity and reduces cardiac fibrosis in a mouse model of chronic *Trypanosoma cruzi* infection

**DOI:** 10.1371/journal.pntd.0007413

**Published:** 2019-05-30

**Authors:** Meagan A. Barry, Leroy Versteeg, Qian Wang, Jeroen Pollet, Bin Zhan, Fabian Gusovsky, Maria Elena Bottazzi, Peter J. Hotez, Kathryn M. Jones

**Affiliations:** 1 Interdepartmental Program in Translational Biology and Molecular Medicine, Baylor College of Medicine, Houston, Texas, United States of America; 2 Medical Scientist Training Program, Baylor College of Medicine, Houston, Texas, United States of America; 3 Section of Tropical Medicine, Department of Pediatrics, Baylor College of Medicine, Houston, Texas, United States of America; 4 National School of Tropical Medicine, Baylor College of Medicine, Houston, Texas, United States of America; 5 Eisai, Inc., Eisai Inc, Andover, Massachusetts, United States of America; 6 Department of Molecular Virology and Microbiology, Baylor College of Medicine, Houston, Texas, United States of America; 7 Department of Biology, Baylor University, Waco, Texas, United States of America; Universidade Federal de Minas Gerais, BRAZIL

## Abstract

Chagas disease, caused by the parasite *Trypanosoma cruzi*, develops into chronic Chagas’ cardiomyopathy in ~30% of infected individuals, characterized by conduction disorders, arrhythmias, heart failure, and even sudden cardiac death. Current anti-parasitic treatments are plagued by significant side effects and poor efficacy in the chronic phase of disease; thus, there is a pressing need for new treatment options. A therapeutic vaccine could bolster the protective T_H_1-mediated immune response, thereby slowing or halting the progression of chronic Chagas’ cardiomyopathy. Prior work in mice has demonstrated therapeutic efficacy of a Tc24 recombinant protein vaccine in the acute phase of Chagas disease. However, it is anticipated that humans will be vaccinated therapeutically when in the chronic phase of disease. This study investigates the therapeutic efficacy of a vaccine prototype containing recombinant protein Tc24, formulated with an emulsion containing the Toll-like receptor 4 agonist E6020 as an immunomodulatory adjuvant in a mouse model of chronic *T*. *cruzi* infection. Among outbred ICR mice vaccinated during chronic *T*. *cruzi* infection, there is a significant increase in the number of animals with undetectable systemic parasitemia (60% of vaccinated mice compared to 0% in the sham vaccine control group), and a two-fold reduction in cardiac fibrosis over the control group. The vaccinated mice produce a robust protective T_H_1-biased immune response to the vaccine, as demonstrated by a significant increase in antigen-specific IFNγ-production, the number of antigen-specific IFNγ-producing cells, and IgG2a antibody titers. Importantly, therapeutic vaccination significantly reduced cardiac fibrosis in chronically infected mice. This is a first study demonstrating therapeutic efficacy of the prototype Tc24 recombinant protein and E6020 stable emulsion vaccine against cardiac fibrosis in a mouse model of chronic *T*. *cruzi* infection.

## Author summary

Chagas disease is a parasitic infection that can cause severe heart disease. Current treatments do not work well and have significant side effects. Because of this, the authors created a new vaccine prototype with the goal that it could be given to infected people to prevent Chagas-associated heart disease. The vaccine contains a manufactured protein identical to a protein in the parasite (called Tc24) as well as a component to help the body produce a protective immune response (a vaccine adjuvant called E6020). The vaccine would boost the body’s natural immune response to the parasite infection, reducing the number of parasites in the body, and protecting the heart. Frequently, people are not diagnosed until later in the infection, because the early (or acute) stage of disease can be mistaken for a common cold. Because of this, it is important to test the vaccine when given in the later (or chronic) stage of infection. The authors tested the vaccine in a mouse model of chronic *T*. *cruzi* infection and found that the vaccinated mice had lower levels of parasites in their body and less damage to their hearts. This research shows promising value of a therapeutic vaccine to prevent Chagas-associated heart disease in a mouse model, with the hope that the same effect could be found in humans one day.

## Introduction

Chagas disease, caused by infection with the protozoan parasite *Trypanosoma cruzi*, is a leading neglected tropical disease globally [1], and the cause of Chagas’ cardiomyopathy, the most common form of non-ischemic cardiomyopathy in Latin America [2]. An estimated 7.2 million people are infected with Chagas disease, with 180,000 new cases occurring annually [3]. Chagas disease is responsible for over $7 billion in lost productivity and health care costs annually [4]. Chagas disease is characterized by two clinically distinct phases of disease: acute and chronic. Acute disease is most often a self-limiting febrile illness, however up to 5% of cases develop severe disease including acute myocarditis, pericardial effusion, and meningoencephalitis, with an estimated 0.5% risk of mortality [5, 6]. In chronic disease, 10–30 years after infection, 30% of patients develop chronic cardiomyopathy [2].

Chronic Chagas’ cardiomyopathy (CCC) clinically presents as conduction disorders and malignant arrhythmias, which can then progress to cardiomyopathy, heart failure, and even sudden cardiac death [7]. The disease is characterized histologically by post-inflammatory myocardial fibrosis [8], and human studies have shown that systemic parasite persistence is associated with disease severity [9, 10]. Clinical progression and survival are poorer in patients with CCC compared to patients with noninflammatory dilated cardiomyopathy, and myocardial fibrosis has been shown to be an independent predictor of adverse outcomes in CCC [11] [12]. The annual mortality of CCC is 4% [13]. In Latin America, an estimated 1.17 million people suffer from CCC [1], and it is the leading cause of cardiovascular death in people aged 30–50 [14].

Current pharmacological treatments (nifurtimox and benznidazole) have inadequate efficacy beyond the acute phase of disease, as demonstrated by the recent BENEFIT trial [15]. Additionally, both drugs have a significant side effect profile in up to 50% of patients, most frequently gastrointestinal distress, cutaneous hypersensitivity reactions, and neurological symptoms [16]. In the quest to develop new therapeutics, vaccines offer an attractive solution. Production of the cytokine IFNγ by CD8^+^ T cells has been correlated with less severe cardiac disease [17]; therefore, a therapeutic vaccine which bolsters the T_H_1-mediated CD8^+^ T cell immune response to the infection, might slow or halt the progression of disease [18]. A therapeutic vaccine would be highly cost effective as demonstrated by economic modeling [19].

A promising antigen target is the *T*. *cruzi* 24kDa flagellar Ca^2+^ binding protein (Tc24) [20]. In humans, recombinant Tc24 is already utilized for serodiagnosis of Chagas disease and to monitor treatment response [21, 22]. Early studies of a DNA vaccine encoding Tc24 have demonstrated therapeutic benefit in mice [23, 24], showing high levels of antigen-specific IFNγ^+^ CD8^+^ cells, and protecting against parasitemia and cardiac pathology [25]. As a recombinant protein vaccine, Tc24 has shown prophylactic efficacy in mice when formulated with the Toll-like receptor 4 (TLR4) agonist monophosphoryl lipid A [26]. More recently, nanoparticle-encapsulated Tc24 with the TLR9 agonist CpG oligodeoxynucleotides has shown therapeutic efficacy in the acute phase of disease [27]. However, little is known about the protective efficacy of a Tc24 recombinant protein vaccine in the chronic stage of Chagas disease.

In this study, we utilize the TLR4 agonist E6020, mixed into a squalene based stable oil-in-water emulsion, to modulate the immune response alongside recombinant Tc24. E6020 is a synthetic lipid A derivative that has reduced pyrogenicity, while maintaining immunogenicity. E6020 has been proposed as a safe and cost-effective vaccine adjuvant [28], and has been demonstrated to be effective in mouse models of a meningococcus vaccine and toxic shock syndrome [29, 30]. E6020 has previously been proposed as an adjuvant for a Chagas disease vaccine [31], and has demonstrated high levels of antigen-specific IFNγ when combined with recombinant Tc24 and protected from blood and tissue parasite burdens in the acute stage of a mouse model of *T*. *cruzi* infection [32, 33]. Here, we investigate the T_H_1-mediated IFNγ^+^ immune response elicited by a Tc24 recombinant protein in a stable emulsion (SE) E6020 vaccine (Tc24+E6020-SE) and the resulting therapeutic efficacy in a mouse model of chronic *T*. *cruzi* infection.

## Results

### Vaccination protects from cardiac pathology and systemic parasitemia

**Fig 1:**
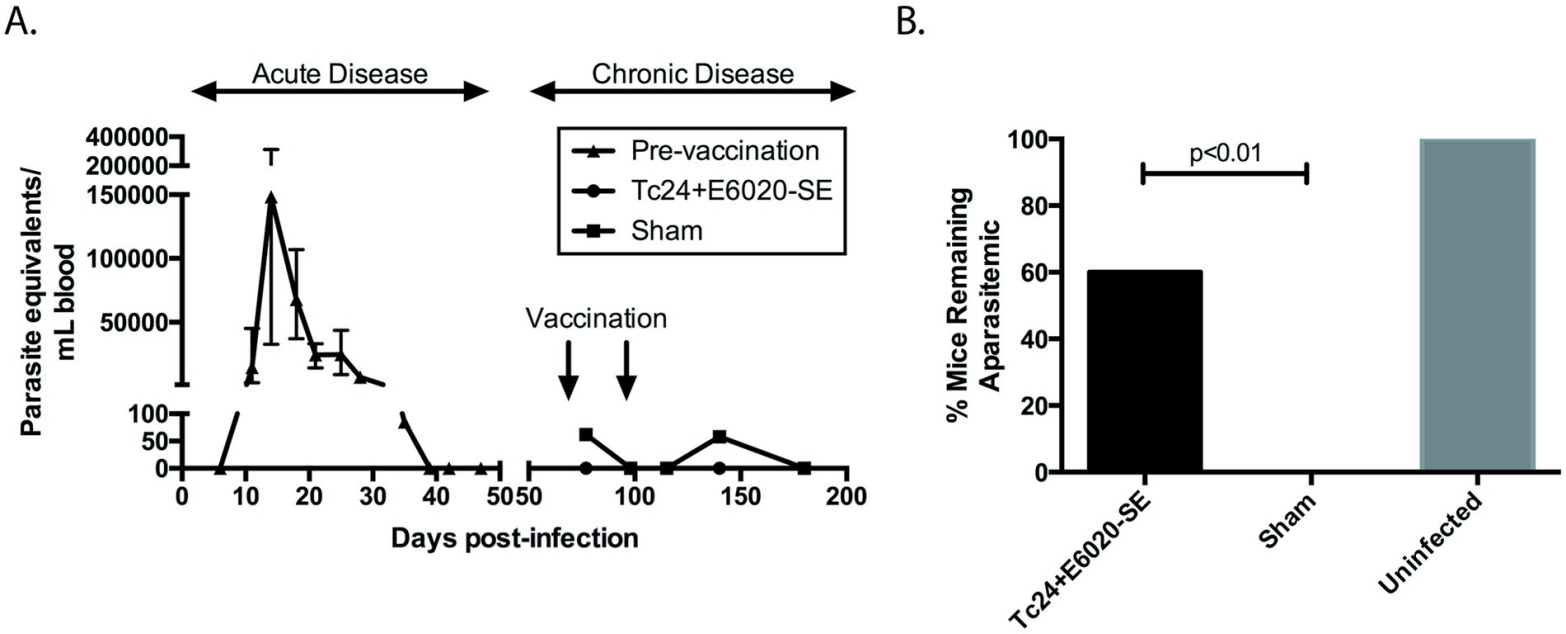
Therapeutic efficacy as measured by systemic parasitemia. ICR mice were infected with *T*. *cruzi* H1 strain parasites. **(A)** Parasitemia was then measured twice weekly by quantitative real-time PCR through the acute phase of infection. Mice were then subsequently immunized in the early chronic stage of infection in a prime boost model at 70 and 98 days post-infection. The Tc24+E6020-SE vaccine was compared to a sham vaccine group and an uninfected control. Parasitemia in the blood was then measured at five times post-vaccination in the chronic phase of infection (at 77, 98, 115, 140, and 180 days post-infection). **(B)** Data shown represents the percentage of mice in each group that remained with undetectable systemic parasitemia at all time points post-vaccination (n = 9–10). Significance between the vaccine and the sham was calculated using Fischer’s exact test.

**Fig 2:**
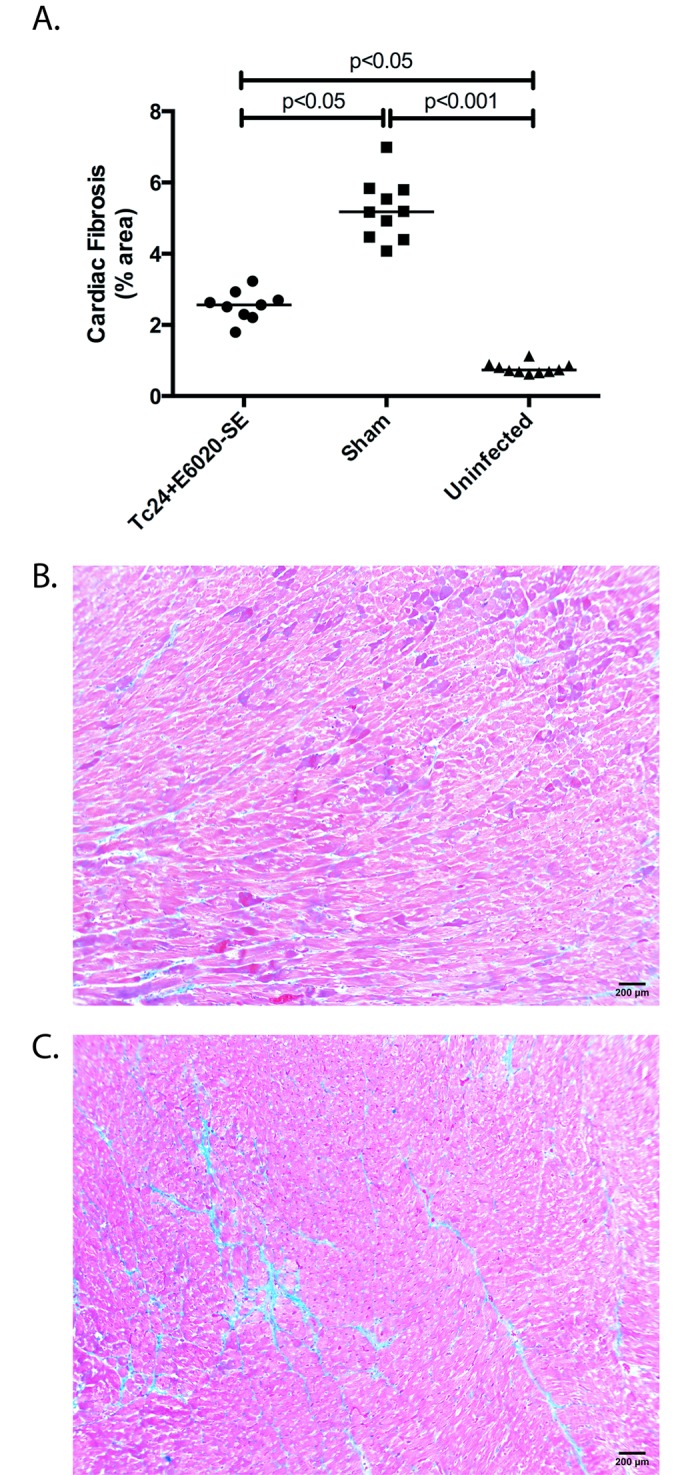
Therapeutic efficacy as measured by cardiac fibrosis. ICR mice were vaccinated with Tc24+E6020-SE vaccine at 70 days post-infection in a prime-boost model, with 4 weeks between prime and boost. The vaccine was compared to a sham vaccine group and an uninfected control. At 180 days post-infection, the hearts were removed, stained with Masson’s Trichrome, and assessed for degree of cardiac fibrosis. **(A)** Quantification of the percentage of area occupied by fibrosis in the cardiac tissue over 30 representative fields of view. Each point represents an individual mouse (n = 9–10); horizontal lines denote median values; significance was calculated by Kruskal-Wallis test with Dunn’s correction for multiple comparisons. Representative sections of a heart from **(B)** a vaccinated mouse, **(C)** a mouse in the sham vaccine group; magnification bar denotes 200 μm.

**Fig 3:**
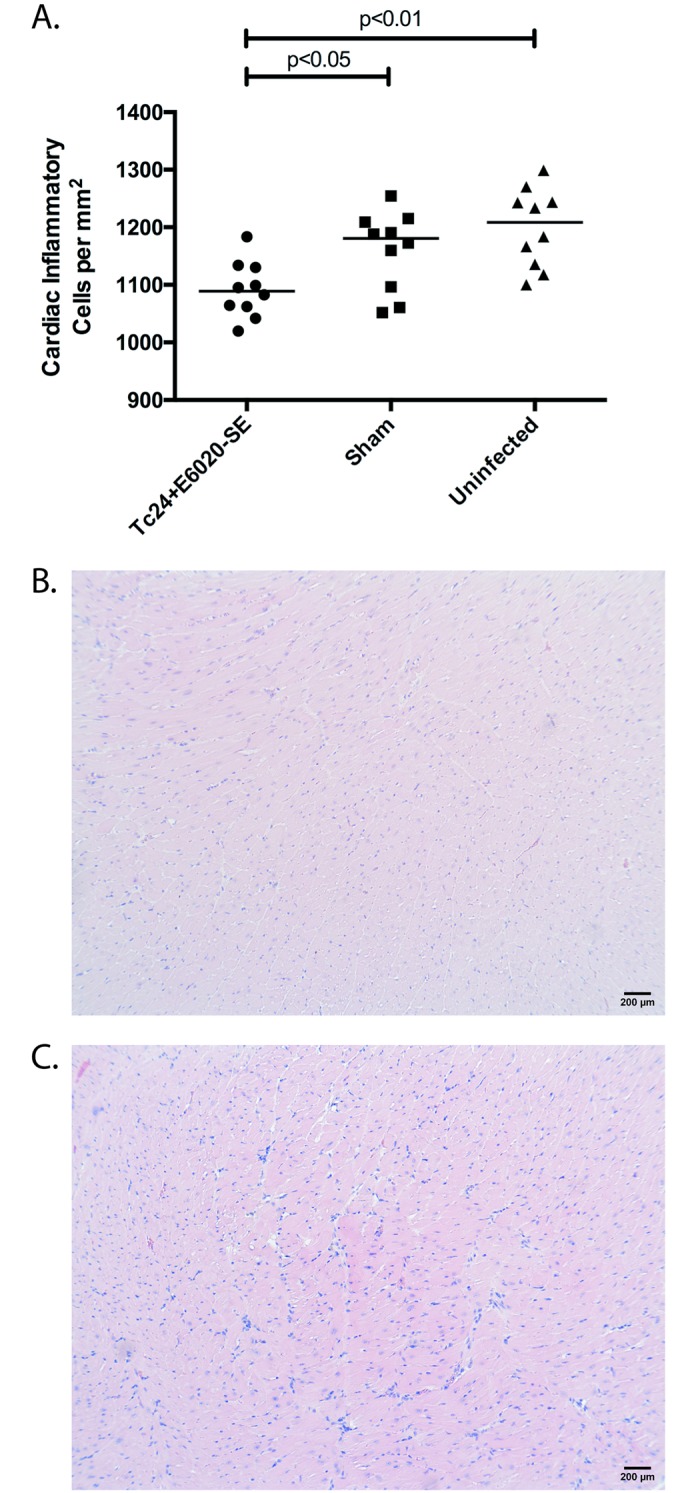
Therapeutic efficacy as measured by cardiac inflammation. ICR mice were vaccinated with Tc24+E6020-SE vaccine at 70 days post-infection in a prime-boost model, with 4 weeks between prime and boost. The vaccine was compared to a sham vaccine group and an uninfected control. At 180 days post-infection, the hearts were removed, stained with H&E, and assessed for inflammation. **(A)** Quantification of the number of nuclei in sections of cardiac tissue over 10 representative fields of view. Each point represents an individual mouse (n = 9–10); horizontal lines denote median values; significance was calculated by Mann-Whitney test comparing each group to the sham vaccinated group. Representative sections of a heart from **(B)** a vaccinated mouse, **(C)** a mouse in the sham vaccine group; magnification bar denotes 200 μm.

In order to evaluate the therapeutic efficacy of the prototype Tc24+E6020-SE vaccine in mice in the chronic stage of *T*. *cruzi* infection, we investigated cardiac pathology and persistence of systemic parasitemia post-vaccination. Mice were infected with *T*. *cruzi* and allowed to progress past the acute phase of disease, characterized by elevated parasitemia resolving by 40 days post-infection (Fig 1A), before vaccination at 70 days post-infection with Tc24+E6020-SE or a sham vaccine. T. *cruzi* infected mice vaccinated with Tc24+E6020-SE vaccine were significantly more likely to have undetectable systemic parasitemia at all measured time points post-vaccination, compared to mice in the sham control group (Fig 1B). Additionally, the therapeutically vaccinated mice had significantly reduced cardiac fibrosis, with an average of 2.5% area of cardiac fibrosis compared to an average of 5% area of cardiac fibrosis in the sham vaccinated control group, as evidenced by analyzing histologic sections of the heart stained for collagen using Masson’s Trichrome (Fig 2). Overall, inflammatory cell infiltrate in the heart was low, approximately 1200 nuclei/mm^2^ in sham vaccinated mice, but there was a slight reduction in vaccinated mice compared to the sham control group when analyzing histologic sections of the heart stained using H&E (Fig 3). We conclude that the Tc24+E6020-SE vaccine is protective against detectable systemic parasitemia and cardiac fibrosis in chronic *T*. *cruzi* infection.

### Tc24+E6020-SE vaccine induces an IFNγ T_H_1-biased immune response

**Fig 4:**
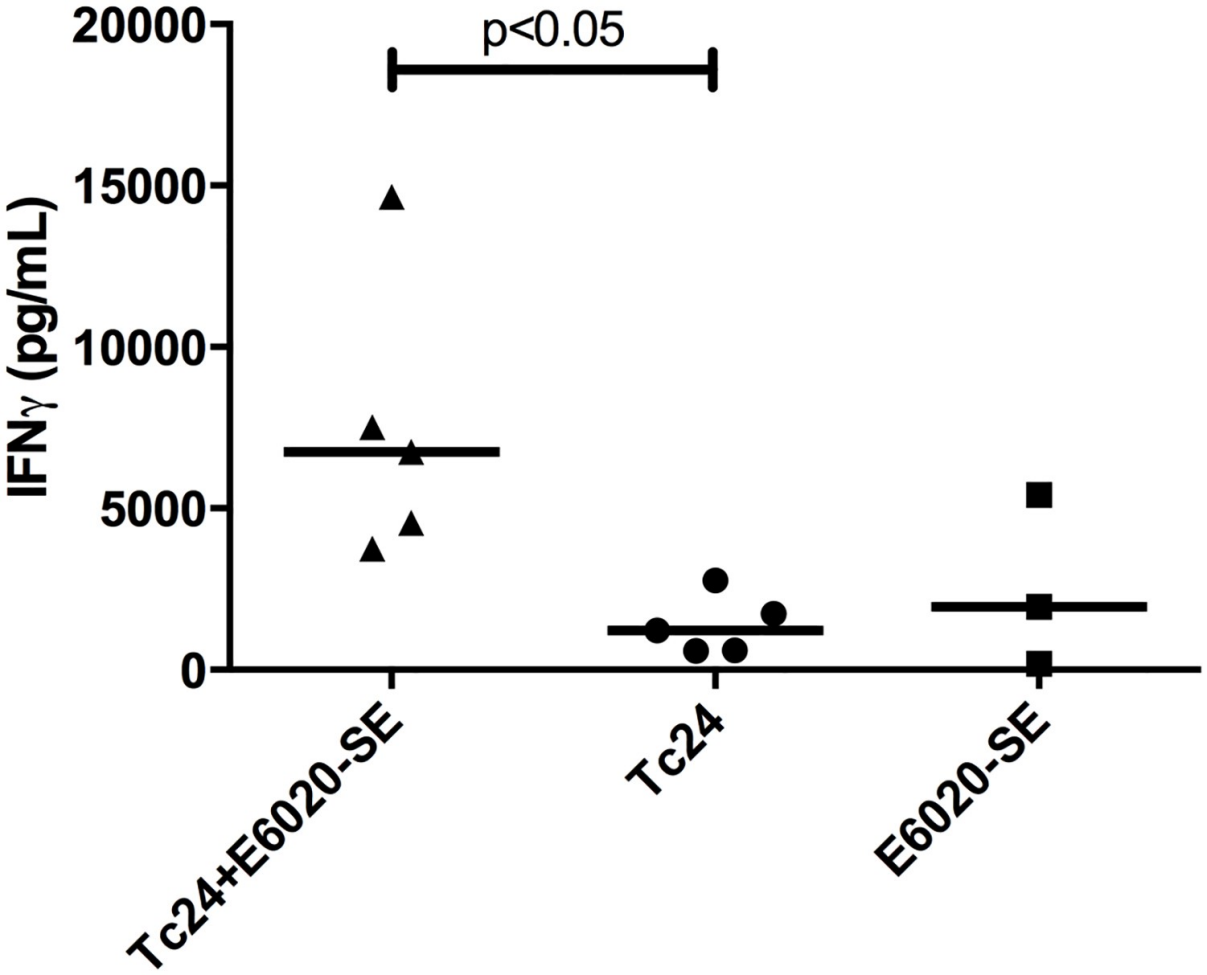
Tc24-specific IFNγ response. Uninfected ICR mice were vaccinated in a prime-boost model, with 2 weeks between prime and boost. The Tc24+E6020-SE vaccine was compared to control groups with only Tc24 or only E6020-SE. Two weeks after the boost vaccination, the Tc24-specific IFNγ producing lymphocyte response was assessed by ELISA. Data shown here is background subtracted; each point represents an individual mouse (n = 9–10); horizontal lines denote median values; significance calculated by Kruskal-Wallis test with Dunn’s correction for multiple comparisons.

**Fig 5:**
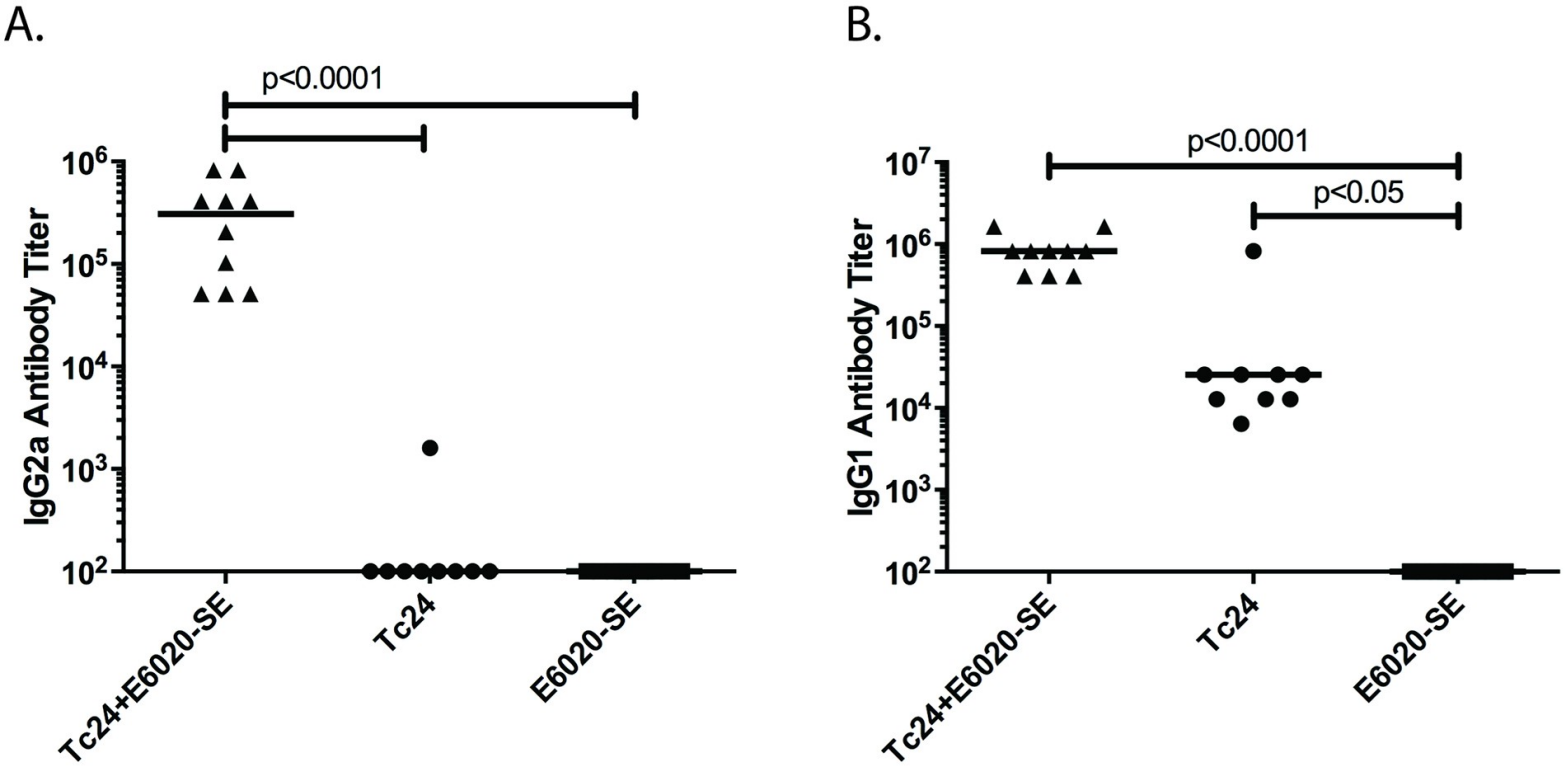
Tc24-specific antibody response. Uninfected ICR mice were vaccinated in a prime-boost model, with 2 weeks between prime and boost. The Tc24+E6020-SE vaccine was compared to control groups with only Tc24 or only E6020-SE. Two weeks after the boost vaccination, the antigen-specific antibody response was assessed by ELISA. **(A)** Tc24-specific IgG2a antibody titer, a T_H_1-associated antibody isotype. **(B)** Tc24-specific IgG1 antibody titer. Each point represents an individual mouse (n = 9–10); horizontal lines denote median values; significance was calculated by one-way ANOVA with Tukey’s correction for multiple comparisons on log transformed data.

To characterize the immune response of the vaccine, both antigen-specific IFNγ production and antibody titers were assessed in uninfected mice immunized with the Tc24+E6020-SE vaccine. The vaccine elicits a greater than 5-fold increase in Tc24-specific secreted IFNγ compared to the Tc24 control (Fig 4). IgG2a, a mouse antibody isotype associated with a T_H_1-bias, is most robust in the vaccine compared to the controls (Fig 5A), further validating the cytokine results. In comparison, both the Tc24+E6020-SE vaccine and the Tc24 control produce a robust IgG1 antibody response (Fig 5B). These results indicate that the Tc24 recombinant protein is capable of producing an antigen-specific immune response, but that the E6020-SE adjuvant is necessary to induce a favorable T_H_1-biasing of the resulting immune response.

### Vaccine induces protective T_H_1-biased immune response in chronic disease

**Fig 6:**
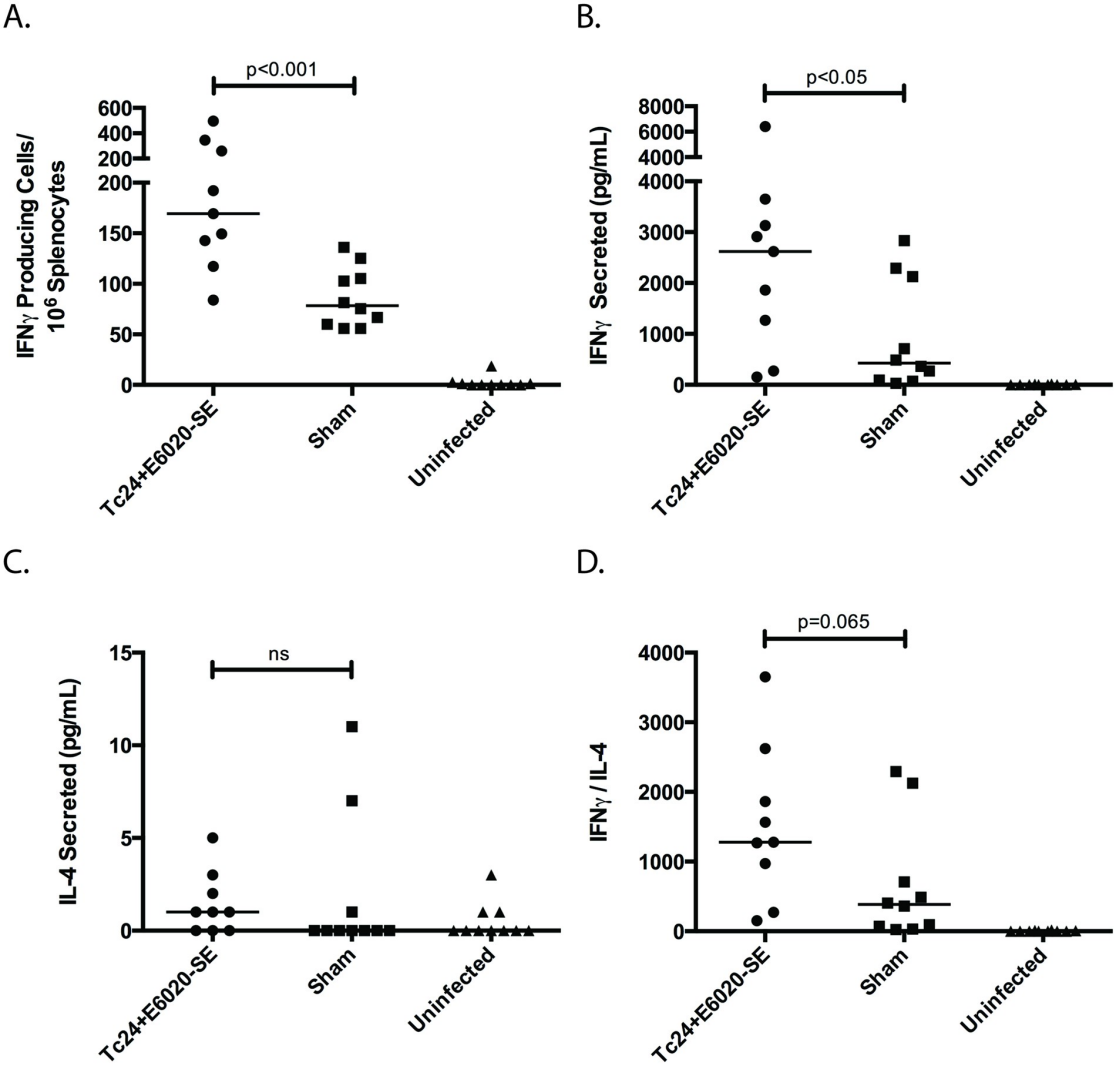
Tc24-specific T_H_1 and T_H_2 cytokine response in *T*. *cruzi* infected mice. ICR mice were vaccinated at 70 days post-infection in a prime-boost model, with 4 weeks between prime and boost. The Tc24+E6020-SE vaccine was compared to a sham vaccine group and an uninfected control. At 180 days post-infection, the antigen-specific cytokine response was assessed. **(A)** Tc24-specific IFNγ producing lymphocytes as assessed by ELISPOT. **(B)** Tc24-specific IFNγ secreted by lymphocytes as assessed by ELISA. **(C)** Tc24-specific IL-4 secreted by lymphocytes as assessed by ELISA. **(D)** T_H_1-biasing of the immune response as determined by the ratio of Tc24-specific secreted IFNγ to IL-4. Data shown here is background subtracted; each point represents an individual mouse (n = 9–10); horizontal lines denote median values; significance shown only between the vaccine and the sham groups and was calculated using the Mann–Whitney U test.

**Fig 7:**
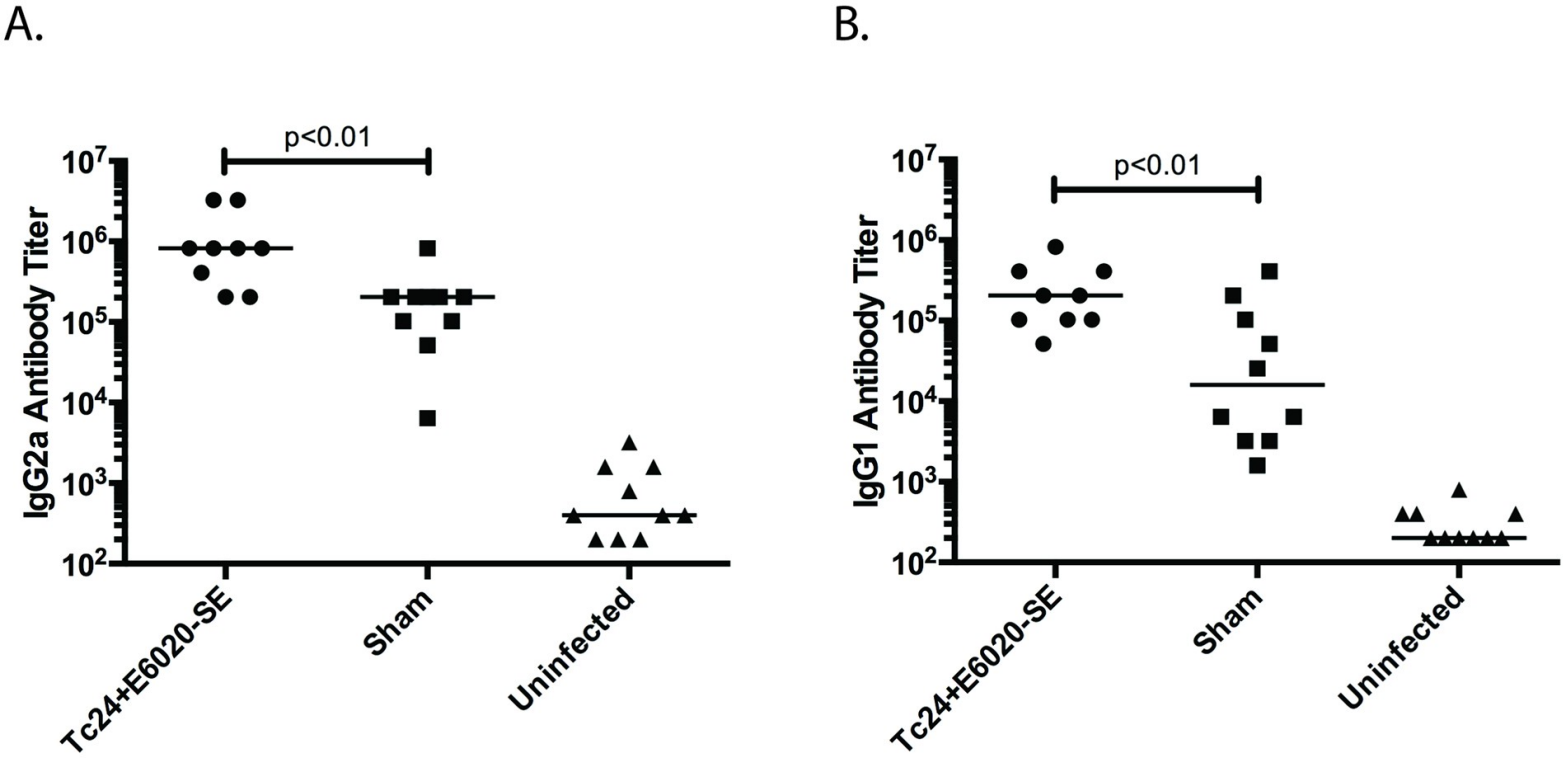
Tc24-specific antibody response in *T*. *cruzi* infected mice. ICR mice were vaccinated at 70 days post-infection in a prime-boost model, with 4 weeks between prime and boost. The Tc24+E6020-SE vaccine was compared to a sham vaccine group and an uninfected control. At 180 days post-infection, the antigen-specific antibody response was assessed by ELISA. **(A)** Tc24-specific IgG2a antibody titer, a TH1-associated antibody isotype. **(B)** Tc24-specific IgG1 antibody titer. Each point represents an individual mouse (n = 9–10); horizontal lines denote median values; significance shown only between the vaccine and the sham groups and was calculated by one-way ANOVA with Tukey’s correction for multiple comparisons on log transformed data.

The protective immune response resulting in therapeutic efficacy was then investigated in mice in the chronic stage of *T*. *cruzi* infection, by characterizing both antigen-specific T_H_1- and T_H_2-associated cytokine production, as well as antibody titers. *T*. *cruzi* infected mice vaccinated with the Tc24+E6020-SE vaccine at 70 days post-infection have a two-fold increase in Tc24-specific IFNγ producing cells compared to the sham control group (Fig 6A). Additionally, they have a six-fold increase in Tc24-specific secreted IFNγ compared to the sham control group (Fig 6B), but no significant difference in Tc24-specific secreted IL-4, a T_H_2-associated cytokine (Fig 6C). Comparing the ratio of IFNγ to IL-4 in order to determine the extent to which the immune response is T_H_1-biased, the Tc24+E6020-SE vaccine produces a three-fold larger IFNγ/IL-4 ratio compared to the sham control group (Fig 6D). Tc24-specific antibody isotype titers further corroborate the cytokine results demonstrating a favorable T_H_1-biased immune response. The Tc24+E6020-SE vaccine produces a significantly greater IgG2a antibody isotype response compared to the sham (Fig 7A), as well as an overall robust IgG1 antibody response (Fig 7B). These results demonstrate that the Tc24+E6020-SE vaccine produces a robust T_H_1-biased immune response in *T*. *cruzi*-infected mice vaccinated in the chronic stage of *T*. *cruzi* infection.

## Discussion

This work is a stride towards the development of a therapeutic Chagas disease vaccine that seeks to prevent or delay the onset of CCC in patients infected with *T*. *cruzi* [31]. Prior studies by this group have shown therapeutic efficacy of Tc24 protein vaccines in a mouse model of acute *T*. *cruzi* infection as evidenced by a reduction in both systemic parasitemia and cardiac parasite burden, and protection from cardiac inflammation [27, 32, 33]. However, it is anticipated that humans will be diagnosed and vaccinated in the chronic stage of disease, as the acute phase of disease is often asymptomatic or a self-limiting febrile illness [31]. Therefore, it is critical to investigate therapeutic vaccine candidates in a model of chronic *T*. *cruzi* infection to more closely replicate the proposed clinical vaccination strategy. Prior studies of a therapeutic Tc24 DNA vaccine administered at 70 days post-infection in the early chronic phase of disease in a mouse model of Chagas disease, similar to the model utilized here, showed improved survival and reduced cardiac inflammation [23]. More recently, an adenovirus-based therapeutic vaccine demonstrated improvements in cardiac histopathology and electrical conduction abnormalities as markers of reduction in CCC disease progression, similar to outcomes measured in this study [34]. However, while precedent exists for licensure of recombinant protein vaccines in humans, no DNA or adenovirus-based vaccines have progressed to market. This is the first study that demonstrates therapeutic efficacy of a Tc24 recombinant protein-based vaccine in a mouse model of chronic *T*. *cruzi* infection.

In this study, we show that a Tc24 recombinant protein vaccine with a stable emulsion containing E6020 as an immunomodulatory adjuvant results in a statistically significant reduction in cardiac fibrosis and inflammation in vaccinated mice compared to mice receiving the sham vaccination. This reduction in cardiac fibrosis is evidence of vaccine protection from chronic Chagas’ cardiomyopathy, as supported by studies that show the cardiac disease is characterized by inflammatory infiltrate and extensive reactive and reparative fibrosis [8, 35, 36]. Additionally, the Tc24+E6020-SE vaccine results in a reduction in detectable systemic parasitemia in vaccinated mice. We propose this reduction and subsequent decrease in parasite-driven tissue damage is one mechanism by which fibrosis is prevented. These findings are supported by studies in human showing systemic parasite persistence is associated with CCC and disease severity [9, 10]. We conclude that the Tc24+E6020-SE vaccine is protective from myocardial fibrosis when used therapeutically in a mouse model of chronic *T*. *cruzi* infection.

A key challenge in the development of this vaccine is the induction of the necessary immune response; unlike most vaccines that rely on an antibody-mediated immune response, a Chagas vaccine will require a protective cell-mediated immune response to be efficacious against the parasite. In this study, to elicit a protective T_H_1-mediated IFNγ^+^ immune response we combined a previously validated protein antigen, Tc24, with a stable emulsion of E6020 as an immunomodulatory adjuvant. In this study, the Tc24+E6020-SE vaccine produces a robust T_H_1-biased immune response, as measured by cytokine and antibody production, in both uninfected ICR mice and ICR mice in the chronic stage of *T*. *cruzi* infection. Our findings of a robust T_H_1-biased immune response are consistent with our previous observations using this adjuvant in other mouse models [32, 33]. In contrast to Aluminum hydroxide vaccine adjuvants (such as Alhydrogel), the adjuvant type utilized in 80% of currently licensed vaccines that produces a predominantly T_H_2-skewed immune response, [37, 38] E6020 preferentially induces a T_H_1-skewed immune response through the TLR4 pathway [39]. It is key to note that chronically infected humans have high serum levels of circulating pro-inflammatory cytokines, including IFNγ, IL-6, TNFα and IL-1β [40], as well as antigen specific pro-inflammatory cells [17]. In pre-clinical models it has been demonstrated that *T*. *cruzi* derived glycoinositolphospholipid (GIPL) is a potent agonist of the TLR4 receptor, enhancing production of TNFα and MIP while increasing neutrophil recruitment, and conferring some resistance to *T*. *cruzi* infection [41]. However, TLR4 receptor engagement by GIPL does not provide long term benefit on disease pathogenesis as wild type TLR4 mice still succumb to infection, although at a later time than TLR4 deficient mice [41]. Additionally, GIPL has been shown to have immunomodulatory effects, downregulating IL-2 production and proliferation of T cells in a reversible manner, since removal of GIPL restores T cell proliferation to baseline levels [42]. Further, GIPL downregulates costimulatory molecule expression on APCs, including macrophages and dendritic cells [43]. These data indicate that while *T*. *cruzi* derived GIPL induces pro-inflammatory responses via TLR4 receptor engagement, it plays a larger role in subverting the immune response. In the face of *T*. *cruzi* derived GIPL being present in chronically infected mice, we hypothesize that the TLR4 agonist E6020 is a more potent stimulator of the TLR4 receptor, similar to LPS which has been shown to induce increased TNFα both locally and systemically, and for a longer duration, when compared to GIPL [41]. E6020 has been shown to have a promising safety profile based on studies in animal models [44]. Precedent exists for utilizing a TLR4 agonist in humans: the licensed vaccines Fendrix, for the prevention of hepatitis B, and Cervarix, the HPV vaccine, both utilize the TLR4 agonist monophosphoryl lipid A [45]. Compared with monophosphoryl lipid A, E6020 has a simplified structure, replacing the typical disaccharide backbone with a simple hexa-acylated acyclic backbone, allowing for faster compound synthesis and improved yield of high-purity material [28, 46].

While small animal models do not mimic all aspects of human Chagas disease, the mouse model described here developed significant cardiac fibrosis, which is a key component of CCC [11, 47]. In the model described in this manuscript mice developed significant cardiac fibrosis, which is a hallmark of chronic determinate disease in humans [35, 48]. Further, it has been shown that up to 72% of patients in the clinically silent chronic indeterminate phase have evidence of cardiac fibrosis [49], thus the mouse model described here does mimic a key component of human disease and can be used to screen novel therapies for efficacy prior to advancing to human studies. One limitation of this model is the overall low level of cardiac inflammation, which is a key finding in human cases of CCC [47]. Here we showed that infected sham vaccinated outbred ICR mice had an average inflammatory infiltrate of approximately 1200 nuclei per mm^2^ tissue (Fig 3), which is very low compared to our prior studies showing approximately 6000 nuclei per mm^2^ tissue in acutely infected inbred BALB/c mice [50]. This difference in cardiac pathology depending on mouse genetic background mimics the differences in cardiac inflammation found by Pereira and colleagues who showed that C3H mice had much greater cardiac inflammation when compared to C57BL/6 mice when both were infected with the Colombian *T*. *cruzi* strain [51]. However, despite the comparatively low cardiac inflammation, infected C57BL/6 mice did develop significant cardiac fibrosis which the authors concluded represented a mild CCC phenotype [51]. In the outbred ICR model of chronic *T*. *cruzi* H1 infection we report in this manuscript, we do see significant cardiac fibrosis where the infected untreated mice had an average of 5.0% fibrotic area compared to 1% in naïve age matched controls (Fig 2), demonstrating that this model does mimic the significant cardiac fibrosis seen in human disease and is useful for evaluating the effect of novel therapies for reducing cardiac fibrosis. Further, this finding of significant cardiac fibrosis in the outbred ICR model of chronic infection supports our previously published finding of significant cardiac fibrosis in acutely infected inbred BALB/c mice (2%) compared to age matched uninfected controls (~0.5%) [52]. Thus, while we predict that our findings of vaccine induced immunogenicity and therapeutic efficacy against cardiac fibrosis in a mouse model of chronic *T*. *cruzi* infection will be translatable to humans, additional studies in other models that develop CCC more characteristic of human disease, such as non-human primates, will be necessary before the vaccine can be moved into clinical trials.

In the quest to develop a therapeutic vaccine against Chagas disease, only limited work has been conducted using therapeutic recombinant protein vaccine prototypes in the chronic stage of *T*. *cruzi* infection. This is the first reported study of a therapeutic vaccine utilizing Tc24 protein and a stable emulsion of E6020 as an immunomodulatory adjuvant in a mouse model of chronic *T*. *cruzi* infection. It is also an important proof of principle study that a vaccine administered in the chronic stage of disease reduces parasitemia and myocardial fibrosis, and may thus slow chronic Chagas’ cardiomyopathy disease progression. Ultimately, a vaccine-linked chemotherapy strategy may be employed in Chagas disease treatment, in which the vaccine is paired with reduced-dose chemotherapy to increase efficacy and reduce side effects [31, 33]. Future studies could elucidate the efficacy of this strategy utilizing the Tc24+E6020-SE vaccine. Additionally, scale-up production of the Tc24 recombinant protein (modified to avoid or limit intermolecular disulfide bond formation and aggregation) suitable for non-human primate or human testing is in process [32, 53]. In conclusion, the Tc24+E6020-SE vaccine demonstrates robust T_H_1-biased immunogenicity, decreased systemic parasitemia, and protection from chronic myocardial fibrosis in a mouse model of chronic *T*. *cruzi* infection.

## Methods

### Ethics statement

All studies were approved by the Institutional Animal Care and Use Committee of Baylor College of Medicine (Protocol AN-5973), Assurance numbers D16-00475 (current) and 3823–01 (previous) and were performed in strict compliance with The Guide for the Care and Use of Laboratory Animals (8^th^ Edition)[54].

### Protein production and vaccine formulation

Recombinant Tc24 protein was expressed in an *E*. *coli* system and purified with Ni-column chromatography as has been described previously. [26, 27] Briefly, codon-optimized DNA encoding full-length Tc24 from a Yucatan H1-strain of *T*. *cruzi* was cloned into a pET41a *E*. *coli* expression vector (EMD Millipore, 70556) with deleted fusion GST (NdeI/XhoI). The resulting plasmid DNA was transformed into BL21(DE3) cells (EMD Millipore, 69450) and induced with IPTG for protein production. The recombinant Tc24 was purified with IMAC Ni-column chromatography (GE Healthcare, Little Chalfont, United Kingdom), and then endotoxin contamination was removed with Q column purification (GE Healthcare, Little Chalfont, United Kingdom).

The synthetically produced TLR4 agonist E6020 was obtained from Eisai Inc., Andover, MA, mixed into a stable oil-in-water emulsion (1mg/mL E6020, 5% Squalene, 0.92% D-α-Tocopherol polyethylene glycol 1000 succinate, 0.76% Span 80) in PBS (E6020-SE). The Tc24 protein sample, also in PBS, and E6020 adjuvant were mixed in a 1:1 ratio at bedside, immediately before injection (Tc24+E6020-SE). For all experiments, 0.1mL of the final formulation was injected subcutaneously. In the study assessing therapeutic efficacy in chronic disease, the Tc24+E6020-SE vaccine contained 100μg Tc24 and 25μg E6020 and the sham contained only PBS. In the immunogenicity study, the Tc24+E6020-SE vaccine contained 25μg Tc24 and 5μg E6020, a Tc24 control contained only 25μg Tc24 and an E6020-SE control contained only 5μg E6020. The sham vaccine group contained only PBS (Corning 21-040-CV).

### Mice, infection, and immunization

Female ICR mice (Taconic Biosciences), aged 6–8 weeks, were used in all experiments. Animal experiments were performed in full compliance with the National Institutes of Health Guide for the Care and Use of Laboratory Animals, 8th edition, under a protocol approved by Baylor College of Medicine’s Institutional Animal Care and Use Committee (IACUC), assurance numbers 3823–01 and D16-00475. Two studies were conducted, one investigating the vaccine efficacy and immunology in mice chronically infected with *T*. *cruzi*, and a second study elucidating the immune response to the vaccine in uninfected mice.

*T*. *cruzi* H1 strain parasites, previously isolated from a human case in Yucatan, Mexico, [23, 55] were maintained by serial passage in mice. To test therapeutic efficacy of the vaccine, naïve mice were infected intraperitoneally with 500 trypomastigotes then subsequently immunized in a prime boost model with vaccine or sham. The mice were first immunized subcutaneously at 70 days post-infection, and then a boost vaccination was administered four weeks later at 98 days post-infection. Mice were sacrificed at 180 days post-infection.

For the immunogenicity study, mice were vaccinated in a prime boost model subcutaneously with vaccine, control groups, or sham, and then a boost vaccination was administered two weeks later. Mice were sacrificed two weeks after the boost vaccination. At the conclusion of both studies, mice were humanely euthanized using ketamine/xylazine-induced deep anesthesia followed by cervical dislocation and then blood, spleens, and hearts were collected. Throughout the studies, all efforts were made to minimize suffering.

### Evaluation of parasitemia

Blood was collected from infected mice semi-weekly through the acute phase of disease and then at five time points post-vaccination in the chronic phase of disease (at 77, 98, 115, 140, and 180 days post-infection). Total DNA was isolated from blood using a DNEasy 96 blood and tissue kit (Qiagen, 69581). Parasitemia was assessed by quantitative real-time PCR as previously described [27]. Briefly, PCR was performed using 10ng purified DNA, TaqMan Fast Advanced Master Mix (Life Technologies, 4444557), and oligonucleotides specific for the satellite region of *T*. *cruzi* nuclear DNA (primers 5’ ASTCGGCTGATCGTTTTCGA 3’ and 5’ AATTCCTCCAAGCAGCGGATA 3’, probe 5’ 6-FAM CACACACTGGACACCAA MGB 3’, Life Technologies, 4304972, 4316032) [56, 57]. Data were normalized to GAPDH (primers 5’ CAATGTGTCCGTCGTGGATCT 3’ and 5’ GTCCTCAGTGTAGCCCAAGATG 3’, probe 5’ 6-FAM CGTGCCGCCTGGAGAAACCTGCC MGB 3’, Life Technologies, 4304972, 4316032) [58], and parasite equivalents were calculated from a standard curve [27, 59, 60].

### Cytokine production

For cytokine production analysis, whole spleens from sacrificed mice were passed through 40μm strainers (BD Biosciences, 352340) to dissociate cells and then red blood cells were lysed with ACK lysis buffer (Lonza, 10-548E). Cells were stained with AOPI Staining Solution and viable cells counted using a Cellometer Auto 2000 automated cell counter (Nexcelom Biosciences, Lawrence, MA). Cells were diluted to appropriate concentrations in 10% FBS, 1X Pen/Strep in RPMI-1640 (all from Corning, 35-011-CV, 30-002-CI, 10-040-CV) for cellular assays.

Antigen-specific cytokine release from splenocytes was quantified by ELISA as previously described [27]. Briefly, 2x10^6^ splenocytes/mL were incubated with 50μg/mL Tc24, 5μg/mL ConA, or media only for 72hrs, 37°C, 5% CO2. Supernatant IFNγ and IL-4 was measured by a sandwich ELISA method using Mouse IFNγ and IL-4 ELISA kits (eBioscience, 88–7314, 88–7044). Cytokine concentrations produced by antigen-specific cells were calculated by background subtraction of the media only stimulated cells.

Antigen-specific IFNγ-producing splenocytes were quantified by ELISpot using an ImmunoSpot kit (Cellular Technology Limited, MIFNG-1M). Briefly, 2.5x10^5^ splenocytes were incubated with 50μg/mL Tc24, 5μg/mL Concanavalin A (ConA, Sigma-Aldrich, C0412), or media only for 24hrs, 37°C, 5% CO2. After the cytokine detection process, spots were analyzed using a CTL-ImmunoSpot S6 Macro Analyzer (Cellular Technology Limited, Shaker Heights, OH). The frequencies of antigen-specific IFNγ-producing cells were calculated by background subtraction of the media only stimulated cells.

### Serum antibody titers

Serum antibodies specific for Tc24 were measured by ELISA as previously described [27]. Briefly, blood samples from mice were allowed to coagulate in Serum-Gel clotting tubes (SARSTEDT, 41.1378.005) and then centrifuged to separate serum per manufacturer’s instructions. Plates (Thermo Scientific, 44-2404-21) were coated with 1.25μg/mL Tc24 in coating solution (KPL, 50-84-00), blocked, and serially diluted serum samples were added. Bound antibody was detected with HRP-conjugated goat anti-mouse IgG1 or IgG2a secondary antibody (LifeSpan Biosciences, LS-C59107, LS-C59112) and the reaction was developed with TMB Substrate (Thermo Scientific, 34021). Titers were recorded as the last dilution above a threshold O.D., calculated by O.D._Avg_+ 3SD of serum from naïve mice.

### Cardiac fibrosis and inflammation

For histopathological analysis, heart tissue fixed in 10% neutral buffered formalin was embedded in paraffin, cut into 5μm sections, and stained with either Masson’s Trichrome or hematoxylin and eosin (H&E) stain. To assess fibrosis representative tissue sections were chosen for each mouse and 30 images were acquired with a Micromaster microscope (Fisher Scientific) and Micron software at 20x magnification. Image analysis was performed using ImageJ software 1.48v (National Institutes of Health, Bethesda, MD). Pixels corresponding to fibrosis were quantified and normalized to total pixels of the sample to assess the percentage of fibrotic area in the cardiac tissue. To assess inflammation, representative tissue sections were chosen for each mouse and 10 images were acquired with an EVOS microscope (EVOS). Image analysis was performed using ImageJ software 1.48v (National Institutes of Health, Bethesda, MD). The number of total nuclei was quantified and normalized to total tissue area analyzed in the cardiac tissue.

### Statistical analysis

Results were analyzed by the Kruskal-Wallis test with Dunn’s correction for multiple comparisons, or, when only two comparison groups were analyzed, Mann–Whitney U test. Results for serum antibody were log-transformed and analyzed by one-way ANOVA with Tukey’s correction for multiple comparisons, as has been previously described [27]. Results for systemic parasitemia were analyzed by Fischer’s exact test. Differences between treatment groups were considered statistically significant if the p-value was less than 0.05. Statistical analysis was performed using Prism software 6.0 (GraphPad Software, La Jolla, CA).
